# Citicoline induces angiogenesis improving survival of vascular/human brain microvessel endothelial cells through pathways involving ERK1/2 and insulin receptor substrate-1

**DOI:** 10.1186/2045-824X-4-20

**Published:** 2012-12-10

**Authors:** Jerzy Krupinski, Manal Abudawood, Sabine Matou-Nasri, Raid Al-Baradie, Eugen Bogdan Petcu, Carlos Justicia, Anna Planas, Donghui Liu, Norma Rovira, Marta Grau-Slevin, Julio Secades, Mark Slevin

**Affiliations:** 1 Cerebrovascular Diseases, Department of Neurology, Hospital Universitari Mútua Terrassa, Terrassa, Barcelona, Spain; 2 Department of Medicine, University of Manchester, Manchester, UK; 3 School of Healthcare Science, Manchester Metropolitan University, Manchester, UK; 4 College of Applied Medical Science, Almajmaáh University, P.O. Box 1405, Almajmaah, 11952, Kingdom of Saudi Arabia; 5 School of Medicine, Centre for Medicine and Oral Health campus, Gold Coast Campus, Griffith University, Queensland, QLD, 4222, Australia; 6 Department of Brain Ischemia and Neurodegeneration, IIBB-CSIC, IDIBAPS, Rossello 161, Barcelona, E-08036, Spain; 7 Cardiovascular Research Center, Hospital de Sant Pau and IIB-Sant Pau, CiberOBN-Instituto de Salud Carlos III, Barcelona, Spain; 8 Ferrer Grupo, Barcelona, Spain

**Keywords:** Citicoline, Angiogenesis, Apoptosis, Stroke, Ischaemia, IRS-1

## Abstract

**Background:**

Citicoline is one of the neuroprotective agents that have been used as a therapy in stroke patients. There is limited published data describing the mechanisms through which it acts.

**Methods:**

We used *in vitro* angiogenesis assays: migration, proliferation, differentiation into tube-like structures in Matrigel™ and spheroid development assays in human brain microvessel endothelial cells (hCMEC/D3). Western blotting was performed on protein extraction from hCMEC/D3 stimulated with citicoline. An analysis of citicoline signalling pathways was previously studied using a Kinexus phospho-protein screening array. A staurosporin/calcium ionophore-induced apoptosis assay was performed by seeding hCMEC/D3 on to glass coverslips in serum poor medium. In a pilot *in vivo* study, transient MCAO in rats was carried out with and without citicoline treatment (1000 mg/Kg) applied at the time of occlusion and subsequently every 3 days until euthanasia (21 days). Vascularity of the stroke-affected regions was examined by immunohistochemistry.

**Results:**

Citicoline presented no mitogenic and chemotactic effects on hCMEC/D3; however, it significantly increased wound recovery, the formation of tube-like structures in Matrigel™ and enhanced spheroid development and sprouting. Citicoline induced the expression of phospho-extracellular-signal regulated kinase (ERK)-1/2. Kinexus assays showed an over-expression of insulin receptor substrate-1 (IRS-1). Knock-down of IRS-1 with targeted siRNA in our hCMEC/D3 inhibited the pro-angiogenic effects of citicoline. The percentage of surviving cells was higher in the presence of citicoline. Citicoline treatment significantly increased the numbers of new, active CD105-positive microvessels following MCAO.

**Conclusions:**

The findings demonstrate both a pro-angiogenic and protective effect of citicoline on hCMEC/D3 *in vitro* and following middle cerebral artery occlusion (MCAO) *in vivo*.

## Background

Citicoline, is an essential component of cell membrane phospholipids, and is one of the neuroprotective agents that have been used as a therapy in stroke patients. It has been extensively tested in many stroke studies and has shown promising results with regard to the reduction of infarct size and improvement of functional recovery. Citicoline (CDP-choline or cytidinediphosphate choline; cytidine 5^′^-diphosphocholine) is a complex organic molecule composed of ribose, pyrophosphate, cytosine and choline [1]. Citicoline has been suggested to provide beneficial recovery and neuroprotective effects in brain traumatic injuries, stroke, brain ageing and neurodegenerative diseases [2,3], although the exact mechanisms through which it operates are not fully understood.

The most recent clinical and pre-clinical updates have been published by Davalos and Secades [4]. In this article, they provide information on basic research studies using animal models, where individual studies have demonstrated for example, increased protection against cognitive impairment in chronic hypoperfused rats [5], as well as a meta-analysis of the effects of citicoline using a systematic review of all the data collected (15 studies on focal ischaemia using the rat model). Here, Giralt et al. [6] showed overall a reduced infarct volume of 30% (transient occlusion) and 25% (permanent occlusion), however disappointingly, there was no improvement in neurological outcome. Finally, Saver et al., [7] performed a meta-analysis using 10 trials of ischaemic/haemorrhagic stroke where the patients were treated with citicoline. Results showed that citicoline treatment was associated with a significant reduction in the frequency of death and disability at long-term follow up with no adverse affects. In all instances, citicoline was administered within 24 h of the stroke occurrence. Interestingly, ‘priming’ of patients with citicoline following transient ischaemic attack (TIA) or minor stroke has not been considered as yet and also, since the mechanism of its action is not fully elucidated, optimal application cannot be determined accurately.

In terms of analysis of the signalling mechanisms associated with citicoline-induced protection, Krupinski et al., [8] showed that citicoline treated animals showed a dramatic reduction in immunoreactive cells for pro-caspases 1, 2, 3, 6 and 8 in the ischemic infarction area when compared with the control group. The number of cells expressing cleaved caspase-3 and nuclear DNA fragmentation in the penumbra area was significantly reduced in animals treated with citicoline. The data suggests that citicoline may protect the ischemic neurons by providing a negative effect on the activation of the caspase apoptotic pathway.

As far as we are aware, no-one has examined the possible beneficial effects of citicoline treatment on revascularization and angiogenesis after stroke. Our preliminary *in vitro* studies have shown that citicoline also protects human brain microvascular endothelial cells (hCMEC/D3) against apoptosis and excitotoxic damage, strongly induces angiogenesis and significantly increases vascularisation in stroke affected regions of rats following MCAO through a signalling pathway involving activation of the insulin-receptor-substrate-1 (IRS-1), suggesting a novel protective mechanism of action and potential clinical application for improvement of tissue reperfusion and neuronal survival after ischaemic stroke.

## Methods

### Cell culture

Human brain micro-vessel endothelial cell (EC) line named hCMEC/D3 was grown in endothelial basal medium-2 (EBM-2) medium supplemented with growth factors and hydrocortisone as described previously [9]. Cells were seeded into T25 flasks pre-coated with 0.1% collagen and maintained in a humidified 5% CO_2_ atmosphere at 37°C. Every three days reaching the confluence, the cells were detached under the enzymatic activity of the trypsin then the cells in suspension were centrifuged for 5 min at 1300 rpm then seeded into new pre-coated T25 flasks. Throughout the study, the cells used were between passages 28 and 38. Cells were routinely cultured as described in our previously published work [10].

### Staurosporin/ionophore-induced apoptosis assay

In this assay, glass coverslips were sterilized in a bath of 100% ethanol for 10–20 min then left to air dry. The coverslips were put in a 24-well plate and pre-coated with 500 μl of 0.1% collagen in acetic acid and then incubated for 1 h_._ hCMEC/D3 were cultured in complete medium at a concentration of 5 × 10^4^ cells/ml on collagen pre-coated coverslips for 4 h-incubation. Then, the medium was replaced with serum-free medium and the cells were incubated. After 24 h incubation, the cells were pre-incubated with 10 μM citicoline for 4 h prior to apoptosis induction. After the pre-incubation with citicoline, apoptosis was induced using calcium ionophore (10 μM/24 h); or staurosporin: 10 μM/4 h (concentrations determined from pilot studies as optimal) or by exposure to oxygen-deprivation (12 h, 1% O_2_; hypoxia confirmed by up-regulation of HIF-1α as determined in our pilot studies). These concentrations have previously been shown to induce apoptosis in about 40-90% of the cell population. For staining, one hour before the termination of the experiment, propidium iodide (PI; 10 μg/ml) was added in each well as an indicator of DNA damage. After 1 h, the medium was discarded and the cells were washed with PBS then fixed with 4% paraformaldehyde for 20 min at room temperature. Subsequently, cells were washed three times with PBS and exposed to 1 μg/ml Hoechst 6024 stain solution diluted in PBS at room temperature for 30 min. Finally, the cells were washed three times with PBS and one drop of FluorSave™ reagent was added on frosted glass slides and the coverslips were put upside down on the drops. An average of six fields at x200 of magnification was photographed per coverslip using an Axivoert fluorescence microscope. The apoptotic index is expressed as the number of apoptotic cells relative to the total number of cells (% apoptotic cells). In this experiment, triplicate wells were run for each condition with controls consisting of untreated cells.

### Angiogenesis assays: cell proliferation

hCMEC/D3 cells were seeded at a concentration of 8 × 10^4^ cells/ml in 500 μl of complete basal medium in each well of 24-well plate. After 4 h, the medium was changed to serum-poor medium (SPM) containing 1% FBS containing different concentrations of citicoline (1 μM, 10 μM and 100 μM; NOTE; pilot experiments were carried out using 1-100 μM citicoline and optimized for the use of 10 μM subsequently as this produced the most prominent responses). After 72 h incubation, cells were washed with PBS and detached with trypsin. Cells were counted in a Coulter counter at least three times for each well. In this experiment, cells were treated in triplicate for each experimental condition. Cell migration (Boyden Chamber: chemotaxis assay)- Inserts of Transwell Costar® porous membranes were coated with 0.1% collagen and left for air dry in a 24-well plate. (Wound recovery)-cells were seeded onto plastic coverslips and when confluent, were scraped with a razor to induce straight lesions. Cells were seeded in 100 μL of serum- poor medium containing 1% FBS at a concentration of 7.3 × 10^4^ cells/ml. The inserts containing hCMEC/D3 were placed in a 24-well plate containing 500 μl of serum poor medium containing 1% FBS and supplemented with either citicoline (10 μM/ml) or FGF-2 (25 ng/ml), used as a positive control. Cells were treated in triplicate for each experimental condition. (Boyden chamber)-After 24 h incubation, the medium was removed from the insert and the cells which had migrated through the pores to the bottom side of the insert were fixed with 4% paraformaldahyde. Cotton swabs were soaked with PBS and used in order to remove the cells that did not migrate. After fixation, the migrated cells were stained with Giemsa (3 minutes). An optical microscope was used to count randomly five microscopic fields from each insert. (Wound healing)-after 24 h the plastic coverslips were fixed in paraformaldehyde and cell migration examined under a microscope at low power. Tube-like structure formation in Matrigel- EC (2 × 10^6^ cells/ml) were mixed with equal volume of growth factor-reduced Matrigel™ containing citicoline (10 mM) or fibroblast growth factor-2 (FGF-2) (25 ng/ml). Any material in the contact with the gel was cold to avoid the gel polymerization. In a 48-well plate, a total amount of 35 μl of this mixture was placed in a tear-like drop in the middle of the well and left to polymerize for 1 h incubation. Then, 0,5 ml of complete medium was added into each well. After 24 h incubation, 4% paraformaldahyde was added to fix the endothelial tube-like structures embedded in the gel. Five areas from each well were counted under optical microscope. Spheroids were produced by inoculation of centrifuged endothelial cells into matrigel with or without addition of citicoline or FGF-2 as a positive control and their development including sprout formation monitored as described above over a period of 7 days. At least 10 sprouts were measured for each condition and the experiments repeated at least 3 times.

### Protein extraction and western blotting

EC (3 × 10^5^ cells/ml; 2 ml) were seeded in complete medium in 6-well plate and incubated for 48 h. Then, the medium was replaced with SPM containing 1% FBS. After 24 h incubation, the cells were stimulated with citicoline at 1-10-50 μM for 10 min then the cells were immediately washed with 1 ml of PBS and gently lysed on ice in 50 μl of ice-cold homogenized lysis buffer (PH 7.2). The cells then were scraped, the cell lysate proteins collected and transferred into 0.5 ml micro-centrifuge tubes. Samples were sonicated four times for 10 seconds each time with 10–15 second intervals on ice to rapture the cells and to shear nuclear DNA. Cell lysates were centrifuged at 20,000 g for 20 min and the protein concentration was determined using the BioRad protein assay.

**Figure 1:**
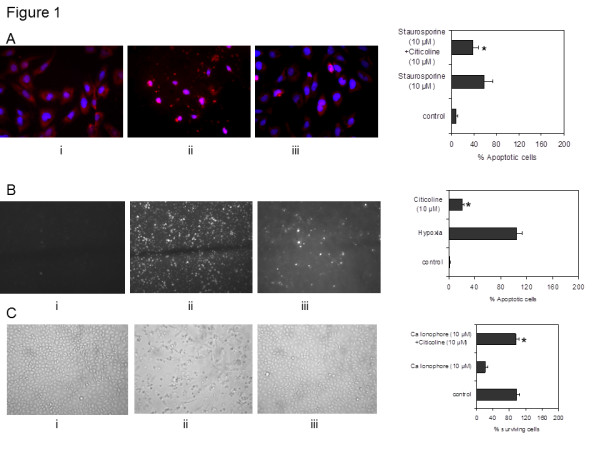
**Effect of citicoline, hypoxia, and the Ca ionophore A23187 on cell death in hCMEC/D3. A**) Shows control hCMEC/D3 without citicoline or staurosporin treatment (i), cells treated with staurosporin (ii; 10 μM), and cells pre-incubated with citicoline for 4 h before apoptosis was induced with 10 μM staurosporin (24 h; iii). The presence of citicoline (10 μM) was sufficient to significantly inhibit cell apoptosis (iii) **B**) Shows control hCMEC/D3 without citicoline or hypoxia treatment (i), cells treated after exposure to hypoxia (ii; 12 h; 1% O_2_) and cells pre-incubated with citicoline for 4 h prior to subjecting to hypoxia (iii). The data shows that citicoline (10 μM) was able to significantly reduce the number of cells undergoing apoptosis (iii). **C**) Shows that citicoline protected the cells against apoptosis induced by calcium ionophore. (i) Shows control cells, (ii) the effect of 10 μM/24 h treatment with Ca ionophore, and (iii), the effect of 4 h pre-treatment with citicoline before addition of 10 μM Ca ionophore. Citicoline significantly protected against apoptosis induced by the ionophore. The bar graphs shows data from one representative experiment carried out in triplicate wells. All experiments were performed three times. Cells were considered apoptotic when cell nuclei demonstrated positive PI/Hoechst staining and apoptotic morphology (Data not shown). For quantification of PI/Hoechst -positive cells, four fields per section were examined at 200-fold magnification. The apoptotic index was calculated using the formula: Apoptotic index = 100 * (number of PI/Hoescht + cell nuclei per field/total number of cell nuclei per field).

In our preliminary un-published studies, we showed that citicoline also strongly induced angiogenesis in our bovine aortic EC (BAEC) and for this reason we performed a phospho-site protein expression screen using Kinex™ antibodies microarray KAM1.3 and performed by Kinexus (Bioinformatics Corporation, Vancouver, Canada). The most relevant phospho-protein expression up- or down-regulated by citicoline were confirmed by Western blotting (IRS-1, HER2 and Histone H2B; see additionally supplied data). Since phosphorylation of IRS-1 was most apparent when translated to our hCMEC/D3 cells, we investigated its role in intracellular signalling in more detail here. Standard siRNA transfection (using lipofectamine) was employed to induce a transient down-regulation of IRS-1 RNA (approximately 85-90%-data not included; Figure 1B and C). After 24 h exposure to siRNA or scrambled sequences, cells were used for analysis of tube-like structure formation described previously. The experiment was repeated twice in triplicate wells and a representative example shown.

### Transient rat MCAO

Four male Wistar rats (Charles River) were used in each group (control and citicoline treated), with a weight of 280-320 g at the beginning of the study. Animals were kept on a 12/12 hours light/darkness cycle, with access to food and water *ad libitum*.

The ischemic lesion was induced by transient (90 minutes) occlusion of the right middle cerebral artery (MCAO), using the intraluminal thread occlusion method, described elsewhere [11], under isofluorane anesthesia. The occlusion period and the successful reperfusion of the right MCA were controlled by continuous recordings of ipsilateral laser-Doppler flowmetry (LDF). Animals were intraperitoneally treated with either citicoline (1000 mg/Kg) or saline. Drugs were daily administered until day 7. The first dose was given 15 minutes before reperfusion. 21 days after occlusion animals were anesthetized and perfused through the heart with heparinized saline, followed by 4% paraformaldehyde. Brains were cryoprotected with 30% sucrose, frozen at −40°C and 8-μm thick coronal sections were cut in a cryostat. 1, 7 and 21 days after occlusion rats were anesthetized and MRI T2-weighted images were acquired at 7 T (Bruker BioSpin), in order to study evolution of lesion volume. There was no mortality in this experimental group. Procedures followed were in accordance with institutional guidelines.

### Immunohistochemistry

Immunohistochemical staining was used to determine the relative numbers of microvessels (anti-CD31) and active vessels (anti-CD105) in control and citicoline treated stroked brain tissue after 21 days (n = 5). The mean number of vessels from five areas of stroke-affected tissue was counted microscopically (× 20) in eight sections per animal. Double immunofluorescence was used to assess the distribution of the phospho-protein IRS-1 in relation to active microvessels in the stroke region (CD105/endoglin mouse monoclonal antibody). After incubation with primary antibodies for 1 h at room temperature (1:100), sections were washed and then incubated with the appropriate secondary antibodies (1:50) − fluorescein isothiocyanate-conjugated sheep anti-mouse IgG (Jackson) or tetramethylrhodamineisothiocyanate-conjugated rabbit anti-goat (Jackson). Images were captured with Nikon 80i Digital Microscope using Nis Elements 3.21 software with multichannel capture option. Negative control slides were included where the primary antibody was replaced with PBS.

### Statistical analysis

All *in vitro* experiments were performed at least three times and the results are expressed as the means ± S.D. Section analysis by immunohistochemistry involved counting the numbers of microvessels from 5 regions within the infarcted tissue and from 3 separate sections (1, 5, and 10 from serial sections) for each condition. Statistical significance was tested by Student’s *t*-test and data were considered significant when p ≤ 0.05.

## Results

### Citicoline protects hCMEC/D3 against cell damage/apoptosis

EC were pre-incubated with 10 μM citicoline for 4 h. Then, the cells were incubated with 1-10 μM staurosporin in DMSO for 4 h and 24 h, respectively. These concentrations were chosen in order to identify appropriate levels of cell damage/apoptosis and were classified as preliminary studies. In these experiments, the numbers of apoptotic cells were approximately 40% and 60% at 10 μM (4 h and 24 h respectively) (data not shown). Therefore, 10 μM/4 h was the chosen treatment in order to evaluate whether citicoline could protect the cells against cell death pathways. Pre-treatment of hCMEC/D3 cells with citicoline significantly decreased the number of damaged/apoptotic cells, determined by counting the numbers of PI-positive nuclei at the end of the experiment, under these conditions (Figure 1A; P < 0.05). Since hypoxia is a key feature of acute ischaemic stroke and a major activator of cell apoptotic pathways, we examined its effect on our EC in the presence of citicoline (10 μM/4 h pre-incubation). Again, the presence of citicoline in the media significantly protected the cells against apoptosis/cell damage (Figure 1B; p < 0.05). Increased expression of and release of Ca^2+^ Kainic acid and glutamate also leads to increased cell death after stroke. Ionophore treatment of EC is known to induce Ca influx and rapid dephosphorylation of eNOS at Thr495 resulting in eNOS activation. For this reason, we exposed our EC to the Ca ionophore A23187 (10 μM/24 h), after pre-treatment with citicoline. Figure 1C shows that the Ca ionophore caused significant reduction in survival of cells after 24 h. However in the presence of citicoline, the effects were reversed and the cells remained attached and morphologically identical to control-untreated cells (p < 0.05). The possible mechanism through which citicoline provides this protection is discussed later in this results section.

#### Citicoline had no mitogenic or chemotactic effect but significantly increased wound recovery, spheroid sprouting and strongly induced endothelial tube-like structure formation in Matrigel

Addition of 10 μM citicoline to hCMEC/D3 had no effect on cell proliferation after 72 h incubation compared with control-untreated cells (data not included). The chemotactic effect of citicoline was measured using the Boyden chamber assay. EC were stimulated with 10 μM citicoline and FGF-2 was used as the positive control. After 24 h incubation, a significant increase of cell migration across the filters induced by FGF-2 was observed whereas citicoline showed no chemotactic effect on the EC, compared to the control (data not included).

**Figure 2:**
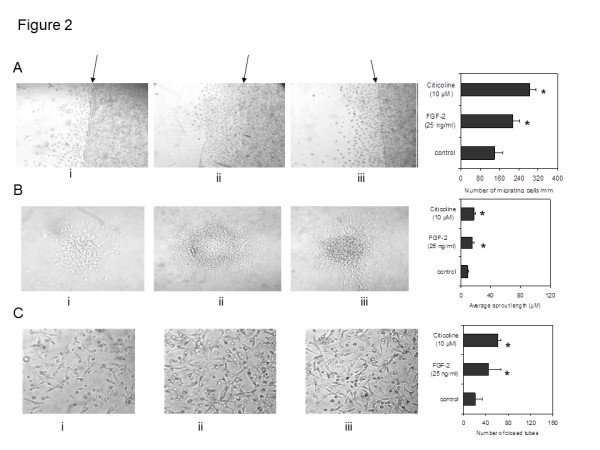
**Effects of citicoline on wound recovery, spheroid sprouting and tube-like structure formation in Matrigel. A**) a significant increase in the number of migrating cells from a scratch wound was seen in hCMEC 24 h after treatment with FGF-2 (positive control; ii) and citicoline (10 μM; p = 0.015; iii). The bar chart represents the mean numbers of migrating cells from triplicate coverslips of control untreated cells, FGF-2 positive control (25 ng/ml), and citicoline treated cells (10 μM). **B**) Shows that addition of citicoline (10 μM) significantly increased the formation and intensity of spheroids as well as migration of cells outwards from the central mass after 7 days incubation (P = 0.005; iii). The bar chart represents the mean sprout length of control spheroids after 7 days, FGF-2 (25 ng/ml), and Citicoline 10 μM. Values are expressed in μM and at least 10 spheroids from each condition were analyzed. **C**) When cells were cultured in Matrigel™ in the presence of citicoline for 24 h, a significant increase in the formation of tube-like structures was observed compared with control cells (p = 0.010; iii). The bar graphs and photomicrographs shows control cells, cells treated with FGF-2 (25 ng/ml), cells treated with citicoline (10 μg/ml). The data illustrates the significant increase in the numbers of closed-loop areas in the presence of citicoline (p = 0.010). Five areas from each well and 3 wells per treatment were analyzed.

However, a significant increase in the wound healing response was seen in hCMEC/D3 after treatment with citicoline (numbers of cells migrated beyond the scratch wound and distance migrated); 10 μM; Figure 2A; (p < 0.05). Similarly, addition of citicoline (10 μM) significantly increased the formation and intensity of spheroids as well as migration of cells outwards from the central mass after 7 days incubation (P < 0.05; Figure 2B).

When the cells were cultured in Matrigel™ in the presence of citicoline for 24 h, a significant increase in the formation of tube-like structures was observed compared with control cells (p < 0.01; Figure 2C). The increase was higher than that observed with FGF-2 a known potent angiogenic factor.

#### Citicoline increased the phosphorylation of phospho-ERK1/2 protein expression and novel proteins detected using a phospho-protein kinexus western array in HCMEC/D3

**Figure 3:**
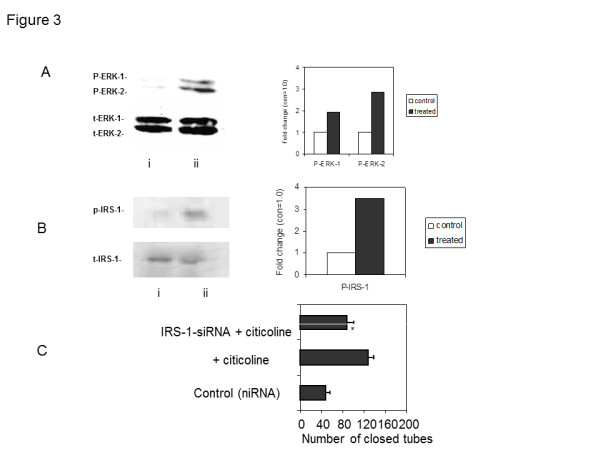
**Citicoline increased expression of p-ERK1/2 and p-IRS-1 in citicoline-treated hCMEC/D3, and treatment with anti-sense siRNA directed against IRS-1 significantly inhibited tube-like structure formation. A** and **B**) Representative Western blots showing the up-regulation of ERK1/2 and IRS-1 phosphorylation after 10 min stimulation of hCMEC/D3 in basal-serum-poor medium. (i) Control cells showed little p-ERK1/2/IRS-1 expression and (ii) after citicoline treatment (10 μM; 10 min) a notable increase was seen. Experiments were performed at least twice and a representative example is shown together with a bar chart showing the fold increase in expression. **C**) hCMEC/D3 treated with anti-sense siRNA directed against IRS-1, for 48 h prior to citicoline treatment (10 μM; 24 h), significantly reduced the ability of citicoline to induce tube-like structure formation in matrigel. Data shown are the result of a single representative experiment performed in triplicate wells. The bar graph illustrates the significant increase in the numbers of closed-loop areas in the presence of citicoline was reduced by approximately 50% in the presence of siRNA to IRS-1 (p = 0.018). Five areas from each well and 3 wells per treatment were analyzed.

Western blotting showed increased expression of phospho-extracellular-signal regulated kinase (ERK1/2), a key angiogenic mitogenic protein in hCMEC/D3 after 10 min stimulation with different concentrations of citicoline (Figure 3A). The screening Kinexus antibody microarray performed by Kinexus on BAEC (Additional file 1: Figure S1) showed a notable increase (> 2 fold) of the phosphorylation of ASK-1, HER2, IRS-1 and Jun and an inhibition (<0.5 fold) for Hsp-70, Integrin alpha4, MEK-1 and Histone H2B proteins (Figure 2; Additional file 1: Figure S1). P-IRS-1 expression was increased in citicoline-treated hCMEC/D3 (Figure 3B).

Since IRS-1 is known to be involved in promotion of angiogenesis of vascular EC [12], we treated our hCMEC/D3 with anti-sense siRNA directed against IRS-1 and following down-regulation of protein expression, observed a significant decrease (approximately 50%) in tube-like-structure formation induced by citicoline alone (Figure 3C), demonstrating that citicoline activates angiogenesis at least partially through a pathway involving IRS-1.

#### Treatment with citicoline significantly increased the numbers of an active (CD105-positive) microvessels in the stroked region 21 days after the infarct

**Figure 4:**
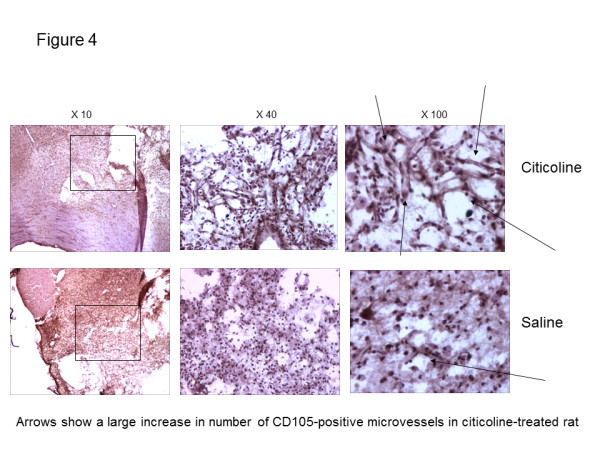
**Immunohistochemical staining for CD105 demonstrated the presence of significantly more active microvessels in stroke regions of rats treated with citicoline following MCAO.** Treatment with citicoline significantly increased the number of CD105-positive microvessels in the peri-infarcted and infarcted regions following transient rat MCAO after 21 days. The figure shows citicoline-treated animals (top row) versus saline treated controls (bottom row). Sections were immunohistochemically stained and developed using DAB (brown). Arrows point to CD105-positively stained microvessels which were consistently more numerous in each animal studied following citicoline treatment (n = 3; p = 0.021).

**Figure 5:**
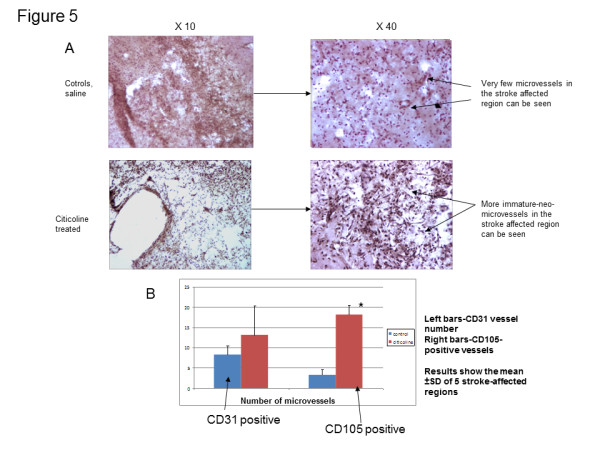
**Immunohistochemical staining for CD31 demonstrated a trend for increased numbers of total microvessels in stroke regions of rats treated with citicoline following MCAO. A**) Treatment with citicoline increased the number of CD31-positive microvessels (total number of vessels but did not reach significance) in the peri-infarcted and infarcted regions following transient rat MCAO after 21 days. The figure shows citicoline-treated animals (top row) versus saline treated controls (bottom row). Sections were immunohistochemically stained and developed using DAB (brown). Arrows point to CD31-positively stained microvessels which were morphologically more immature (thinner walls and less pericyte coverage) present in larger numbers in each animal studied following citicoline treatment (n = 3). **B**) Bar chart showing the increase in CD31 positive and CD105 positive vessels stained in sections (5 analyzed per animal) from rats following transient MCAO and treatment with citicoline as described in the methods section (n = 3 in each group).

Treatment of rats with citicoline (1000 mg/Kg) intraperitoneally prior to reperfusion and daily until euthanasia at day 21 post-infarct, resulted in a notable but not-significant increase in CD31-positive and a significant increase in CD105-positive (active) microvessels in the peri-infarcted and infarcted regions, measured in eight histologically processed serial sections from each rat at the focus of the infarcted cerebral cortex territory (5 areas at x 20 magnification) and compared with saline treated controls (Figures 4, 5). Citicoline treatment had no effect on the total infarct volume over this treatment time.

#### IHC and Double immuno-fluorescent staining demonstrated strong co-localization of CD105-positive neovessels in the peri-infarcted region and p-IRS-1

**Figure 6:**
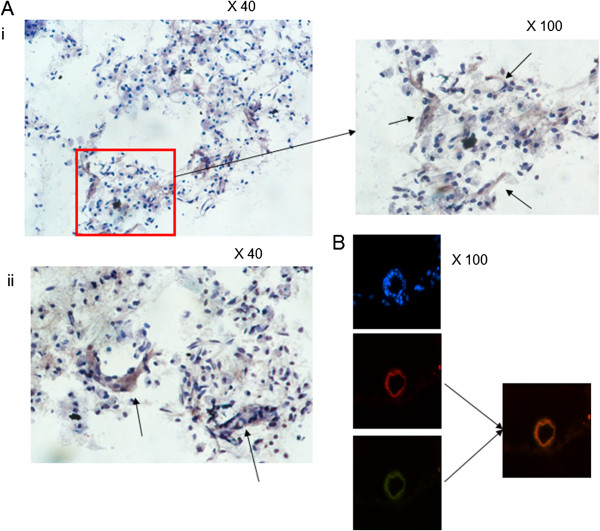
**Co-localization of CD105 (TRITC) and p-IRS-1 was seen in microvessels of citicoline-treated animals in stroke regions following MCAO. Ai** and **ii**) IHC staining of citicoline-treated rats (n = 5) demonstrated a notable increase in the staining of stroke-associated microvessels with p-IRS-1 (DAB; brown). Representative staining is shown from one animal at magnifications of x 40 and x 100. Arrows represent positively stained vessels. **B**) IF analysis demonstrated co-localization of CD105 (TRITC) and p-IRS-1 (FITC) in some of these vessels. In all experiments, control sections were used where the primary antibody was replaced by PBS.

IHC and Immunofluorescent labeling showed increased expression of p-IRS-1 (Figure 6Ai and ii) and co-localization of p-IRS-1 and CD105-positive microvessels in the infarcted region of animals 21 days after the initial MCAO and following citicoline treatment (Figure 6B). Almost no staining was seen in control, untreated animals (data not included).

## Discussion

In this study, we have, for the first time, demonstrated both a vascular protective, and pro-angiogenic effect of citicoline using *in vivo* and *in vitro* models. Here we show the strong protective effect of citicoline on brain microvascular EC, demonstrated by the ability of citicoline to protect against either calcium ionophore or hypoxia-induced cell damage/apoptosis identified by nuclear uptake of propidium iodide. Previous studies have shown that citicoline was able to protect motor neurons of organotypic rat lumbar spinal cord cultures, from apoptosis following administration of the excitotoxic agent DL-threo-β-hydroxyaspartate (a glutamate analogue) [13]. Similarly, citicoline with or without hypothermia, protected neurons against apoptosis in a rat model of transient focal MCAO through a mechanism including the modulation of Bcl-2, Bax and caspase-9 [14]. In other cell types/sources, citicoline protected against cell damage from Kainic acid in retinal neurons [15] and indirectly, may protect lung adenocarcinoma cells (A549) against apoptosis [16,17]. No studies until now have been carried out using EC. Our data suggests a strong protective effect against the damaging process of excitotoxicity and hypoxia, similar to that experienced after acute ischaemic stroke. In regard to the possible mechanism? Our kinexus phospho-protein screen identified a tenfold reduction in expression of Histone H2B (serine 14). Phosphorylation of histone H2B at serine 14 (S14), a posttranslational modification required for nuclear condensation, correlates with cells undergoing programmed cell death in vertebrates [18]. The authors of this paper also identified a 34 kDa apoptosis-induced H2B kinase as caspase-cleaved Mst1 (mammalian sterile twenty) kinase. Mst1 can phosphorylate H2B at S14 *in vitro* and *in vivo*, and the onset of H2B S14 phosphorylation is dependent upon cleavage of Mst1 by caspase-3. These data reveal a histone modification that is uniquely associated with apoptotic chromatin and provide insights into a previously unrecognized physiological substrate for Mst1 kinase. Further experiments are needed to confirm that these findings represent a key novel mechanistic pathway for EC protection associated with citicoline treatment.

In addition, although citicoline had no effect on the chemotaxis of HBMEC/D3 determined by using the Boyden chamber method it significantly increased the number of migrating cells in the scratch wound healing assay. Citicoline also significantly increased the formation of tube-like structure in Matrigel producing a stronger effect than the known mitogenic factor FGF-2 (p <0.05). Although citicoline had no mitogenic and chemotactic effects on EC, it had a significant effect on cell differentiation and migration which are two of the key steps of the angiogenic process. This may be an extremely valuable novel finding in regard to understanding the potential mechanisms through which citicoline treatment results in patient recovery, since both protection of EC and induction and maintenance of angiogenesis is key to both short-term and chronic re-vascularization after stroke impacting indirectly but significantly also on neuronal survival and re-integration [19].

Western blotting demonstrated that citicoline induced pERK1/2 expression, a key mitogenic signalling protein known to be involved in angiogenesis and generally stimulated by growth factors through interaction with their receptors [20]. This data demonstrated the potential of citicoline to activate intra-cellular signal transduction pathways and induce phosphorylation of down-stream angiogenic molecules; hence we investigated this ability in more detail by analysis of the Kinexus-phospho-protein Western screen following treatment of vascular EC with citicoline. Interestingly, treatment with citicoline modified the expression of only several of the >500 proteins on the array showing a degree of specificity. IRS-1 and Her2 were both phosphorylated in the presence of citicoline. These two proteins have not been implicated in stroke recovery pathways or stroke angiogenesis until now.

Here, we went on to demonstrate the importance of IRS-1 in mediating the angiogenic effects of citicoline *in vitro*, and further showed localization of p-IRS-1 in the vascular regions of peri-infarcted tissue of animals 21 days after MCAO but only following treatment with citicoline. The vessels were nearly always thin-walled neo-vessels with minimal or no pericyte coverage and CD105-positive suggesting dynamic activity and contribution to the re-vascularisation process.

Only recently, IRS-1 over-expression was attributed to increased angiogenesis in human EC in association with increased Akt and VEGF-A expression [12], whilst *in vivo*, antisense IRS-1 sequences delivered by sub-conjunctival injection inhibited rat corneal neovascularisation [21], and when delivered by means of eye-drops (GS-101) were found to be tolerable in a phase-1 clinical trial and may be sufficient to prevent neovascularisation in disease such as retinopathy and neovascular glaucoma [22]. Therefore, IRS-1 represents a potent modulator of pro-angiogenic signalling cascades in vascular EC and as such, since we have shown both *in vitro*, and in the rat model of temporary MCAO that citicoline induces phosphorylation of IRS-1 and concomitant EC activation and increased vascularisation, this could be a key novel mechanism of action of citicoline.

A further interesting finding was the up-regulation of HER2 by citicoline in our vascular ECs *in vitro*. Although we have not investigated this in more detail within this piece of work or in our hCMEC, HER2 has previously been shown to be important in promotion of angiogenesis and concomitant tumour breast tumour growth [23], and is strongly implicated in activation of intracellular signalling pathways increasing the expression of VEGF and IL-8 [24]. Therefore, further investigation is warranted to determine its importance in the induction of citicoline-associated angiogenesis and vascularisation after ischaemic stroke.

## Conclusion

In conclusion, citicoline induces angiogenesis and improves survival of human brain microvessel endothelial cells through pathways involving p-ERK1/2, and IRS-1 and it is probable that other novel signalling intermediates are also involved including Histone H2B and HER2 (more studies are needed to confirm this) and therefore following chronic treatment, its beneficial effects after stroke may in part be due to revascularization. Based on our findings, optimization of its therapeutic use to include vascular tissue regeneration should be re-considered.

## Competing interests

The authors declare that they have no competing interests.

## Authors’ contributions

JK, MS and JS designed the experimental protocols/project management and drafted the manuscript; MA, SM and DL performed the experimental work *in vitro*; CA and JP performed the *in vivo* studies; RA, NR, MG and EP helped to design the study and prepare the manuscript. All authors read and approved the final manuscript.

## Disclosures

Authors have no additional disclosures.

## Sources of funding

This study was supported from research grant from Ferrer International.

## Supplementary Material

Additional file 1 **Figure S1.** Kinexus bar chart. Click here for file
