# Supplementary figures and images for: Citicoline induces angiogenesis improving survival of vascular/human brain microvessel endothelial cells through pathways involving ERK1/2 and insulin receptor substrate-1
Source: Vasc Cell. 2012 Dec 10;4:20. doi: 10.1186/2045-824X-4-20 (PMC3554547; doi:10.1186/2045-824X-4-20)

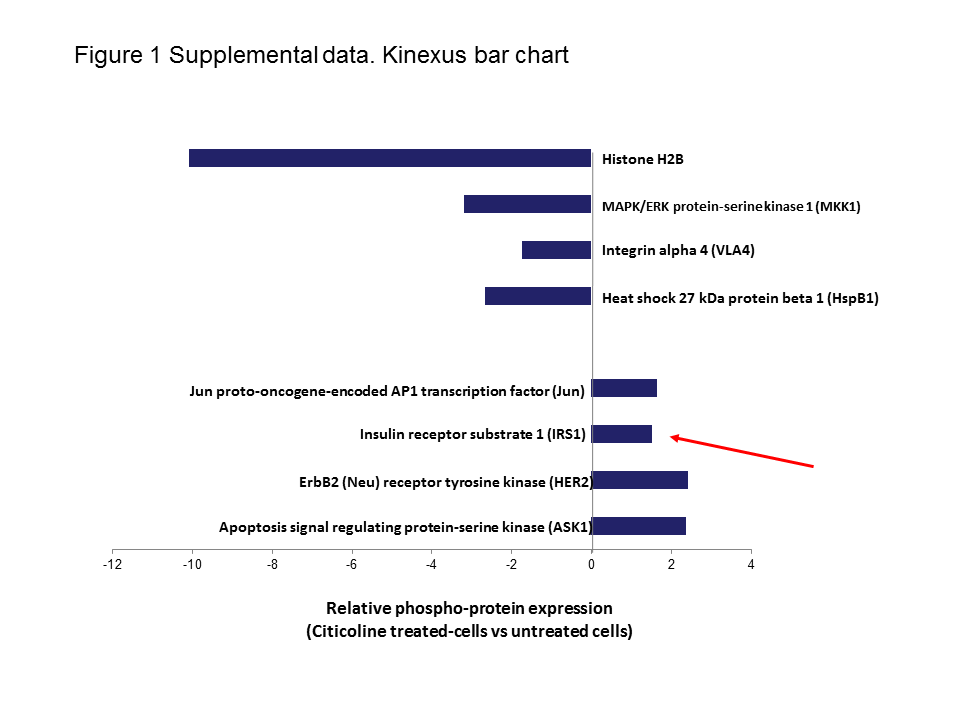

Supplement: Additional file 1 — Figure S1. Kinexus bar chart. [file 2045-824X-4-20-S1.tiff]
